# Ptychographic phase retrieval via a deep-learning-assisted iterative algorithm

**DOI:** 10.1107/S1600576724006897

**Published:** 2024-08-19

**Authors:** Koki Yamada, Natsuki Akaishi, Kohei Yatabe, Yuki Takayama

**Affiliations:** a Department of Electrical Engineering and Computer Science Tokyo University of Agriculture and Technology 2-24-16 Naka-cho, Koganei Tokyo Japan; b International Center for Synchrotron Radiation Innovation Smart, Tohoku University, 468-1 Aoba-ku, Sendai, Japan; c Graduate School of Agricultural Science, Tohoku University, 468-1 Aoba-ku, Sendai, Japan; d Research Center for Green X-Tech, Green Goals Initiative, Tohoku University, 6-6 Aoba-ku, Sendai, Japan; e RIKEN SPring-8 Center, 1-1-1 Kohto, Sayo, Sayo-gun, Hyogo, Japan; SLAC National Accelerator Laboratory, Menlo Park, USA

**Keywords:** hard X-ray ptychography, phase retrieval, deep neural networks, formula-driven supervised learning

## Abstract

Ptychography is a powerful computational imaging technique with microscopic imaging capability and adaptability to various specimens. To obtain an imaging result, it requires a phase-retrieval algorithm whose performance directly determines the imaging quality. Recently, deep neural network (DNN)-based phase retrieval has been proposed to improve the imaging quality from the ordinary model-based iterative algorithms. However, the DNN-based methods have some limitations because of the sensitivity to changes in experimental conditions and the difficulty of collecting enough measured specimen images for training the DNN. To overcome these limitations, a ptychographic phase-retrieval algorithm that combines model-based and DNN-based approaches is proposed. This method exploits a DNN-based denoiser to assist an iterative algorithm like ePIE in finding better reconstruction images. This combination of DNN and iterative algorithms allows the measurement model to be explicitly incorporated into the DNN-based approach, improving its robustness to changes in experimental conditions. Furthermore, to circumvent the difficulty of collecting the training data, it is proposed that the DNN-based denoiser be trained without using actual measured specimen images but using a formula-driven supervised approach that systemically generates synthetic images. In experiments using simulation based on a hard X-ray ptychographic measurement system, the imaging capability of the proposed method was evaluated by comparing it with ePIE and rPIE. These results demonstrated that the proposed method was able to reconstruct higher-spatial-resolution images with half the number of iterations required by ePIE and rPIE, even for data with low illumination intensity. Also, the proposed method was shown to be robust to its hyperparameters. In addition, the proposed method was applied to ptychographic datasets of a Simens star chart and ink toner particles measured at SPring-8 BL24XU, which confirmed that it can successfully reconstruct images from measurement scans with a lower overlap ratio of the illumination regions than is required by ePIE and rPIE.

## Abstract 2

This paper proposes a ptychographic phase-retrieval algorithm combined with a deep neural network (DNN). The proposed method allows the measurement model to be explicitly incorporated into the DNN-based approach, improving imaging capability and robustness to changes in experimental conditions.

## 1. Introduction

Ptychography is a computational imaging technique for microscopic observation using a coherent beam such as visible light, X-rays or electrons (Rodenburg & Faulkner, 2004 ▸). While the spatial resolution of conventional lens imaging is limited by the characteristics of the lens optics, ptychography can overcome such limitations; for example, sub-10 nm resolutions have been achieved in both hard and soft X-ray regions (Deng *et al.*, 2019 ▸; Sun *et al.*, 2021 ▸), and the possibility to attain such high spatial resolution in the tender X-ray region was recently demonstrated using the new 3 GeV high-brilliance synchrotron radiation facility NanoTerasu (Ishiguro *et al.*, 2024 ▸). Ptychography has gained much attention for its capability of imaging electron densities, chemical states, magnetic structures, crystal orientations, strain fields *etc*. (Shi *et al.*, 2019 ▸; Gao *et al.*, 2020 ▸; Uematsu *et al.*, 2021 ▸), with high spatial resolution and adaptability to different types of radiation in various fields, such as materials (Grote *et al.*, 2022 ▸; Uematsu *et al.*, 2021 ▸; Gao *et al.*, 2020 ▸; Pattammattel *et al.*, 2020 ▸) and biological sciences (Polo *et al.*, 2020 ▸; Suzuki *et al.*, 2016 ▸; Shahmoradian *et al.*, 2017 ▸; Deng *et al.*, 2018 ▸). Ptychographic measurement involves illuminating a specimen (the object) with a coherent beam (the probe) and scanning the object with overlapping intervals to obtain the resulting diffraction intensity patterns. These observations contain information about the interaction between the object and incident radiation, encoding the microscopic structure of the object. In ptychography, a computational algorithm extracts the illumination wavefield and the complex-valued refractive index distribution (*i.e.* phase and absorption contrast) of the object from the observed data. This algorithm is often called phase retrieval.

The imaging quality in ptychography generally can be improved by increasing the exposure time (illumination intensity) and/or the number of scan positions (overlap ratio of the illuminated regions). However, such attempts cannot be applied to radiation-sensitive specimens like biological samples (Suzuki *et al.*, 2016 ▸; Zhou *et al.*, 2020 ▸) and polymeric materials (Wu *et al.*, 2018 ▸; De Caro *et al.*, 2016 ▸). In computed tomography, spectroscopic measurements or time-resolved *in situ* observations, it is a challenge to perform multiple measurements of identical samples within the limitation in the tolerable irradiation dose. Consequently, the reconstructed images often have low contrast and suffer from noise. For this reason, the phase-retrieval algorithm must be robust under conditions of low illumination intensity and/or where scans have a low overlap ratio. In addition to this requirement, the algorithm should have a reasonable computational time and be easily adjusted to various specimens and experimental conditions to give feedback on imaging results to the experiments within the limited beamtime.

Many phase-retrieval algorithms have been developed for ptychography. They can be categorized into model-based and deep neural network (DNN)-based approaches. Model-based approaches formulate an optimization problem from the measurement model and solve it by iterative updates based on alternating optimization. Examples of such model-based phase-retrieval algorithms include the conjugate gradient method (Guizar-Sicairos & Fienup, 2008 ▸), the extended ptychographic iterative engine (ePIE) (Maiden & Rodenburg, 2009 ▸), the regularized PIE (rPIE) (Maiden *et al.*, 2017 ▸), the difference map (DM) (Thibault *et al.*, 2009 ▸), relaxed averaged alternating reflections (RAAR) (Luke, 2005 ▸; Marchesini *et al.*, 2016 ▸), the maximum likelihood estimation method (Thibault & Guizar-Sicairos, 2012 ▸) and the proximal splitting algorithm (Hesse *et al.*, 2015 ▸; Chang *et al.*, 2019*a* ▸). Some advanced methods address noise problems, such as camera readout noise and parasitic scattering noise, to realize image reconstruction robust to noise (Yatabe & Takayama, 2022 ▸; Seifert *et al.*, 2023 ▸; Chang *et al.*, 2019*b* ▸). Model-based approaches often incorporate regularization and constraints that reflect prior knowledge into optimization to improve imaging quality. This strategy can realize successful imaging even when the number of observations is insufficient and/or the illumination intensity is low. However, the regularizer must be designed manually, and its proper design is often difficult. This strategy also requires tuning of the hyperparameters associated with the regularizer.

On the other hand, DNN-based phase retrieval can successfully reconstruct images without manually designing regularizers thanks to the data-driven training of the DNN using a large amount of data (Barbastathis *et al.*, 2019 ▸; Bostan *et al.*, 2020 ▸; Nguyen *et al.*, 2018 ▸; Rivenson *et al.*, 2018 ▸; Sinha *et al.*, 2017 ▸; Li *et al.*, 2018 ▸; Cherukara *et al.*, 2020 ▸; Hoidn *et al.*, 2023 ▸). DNN-based approaches exploit the end-to-end learning framework, *i.e.* the trained DNN directly maps observed intensity data to phase. These approaches have achieved state-of-the-art performance in X-ray ptychography (Cherukara *et al.*, 2020 ▸; Hoidn *et al.*, 2023 ▸), Fourier ptychography (Nguyen *et al.*, 2018 ▸), holography (Rivenson *et al.*, 2018 ▸) and computational imaging (Sinha *et al.*, 2017 ▸; Li *et al.*, 2018 ▸). However, DNN-based phase retrieval has some limitations. The first limitation is that DNN-based phase retrieval is sensitive to changes in the experimental conditions and/or specimens (Jo *et al.*, 2019 ▸). Since similarity between the training dataset and the actual observed data is crucial for DNN to work well, DNN-based phase retrieval often fails to reconstruct images when applied to data acquired with an experimental setting that is not included in the training dataset. The second limitation is the difficulty of collecting training datasets. While training of a DNN requires a large amount of data, it is not easy to measure many types of specimens with a variety of experimental conditions due to the measurement costs of ptychography. The difficulty of collecting data was circumvented by a physics-constrained unsupervised deep learning approach (Hoidn *et al.*, 2023 ▸), but this method cannot estimate the probe function, unlike model-based approaches.

In this paper, we propose a ptychographic phase-retrieval algorithm to overcome the above limitations. The proposed method combines model-based and DNN-based approaches by inserting a DNN-based denoiser into an iterative algorithm derived from the measurement model. In the iterative algorithm, the inserted DNN refines the image at each iteration, leading to a higher-spatial-resolution image with fewer iterations than are required by the conventional model-based algorithms. The proposed method is not constructed by heuristically incorporating a deep denoiser into an existing method but is derived from the optimization problem with a fixed-point constraint.

Since the proposed method explicitly incorporates the measurement model, one can easily address changes in specimens and experimental conditions by adjusting the measurement model. This is the advantage of the proposed method over conventional end-to-end DNN approaches that cannot address such changes because they require reconstruction of the dataset and retraining of the DNN.

In addition, to circumvent the difficulty of collecting a large number of actual measured images for training the DNN, we propose to train it using a formula-driven supervised learning (FDSL) technique (Baradad *et al.*, 2021 ▸) which can train the DNN with systematically generated synthetic training datasets.

In experiments using simulation based on a hard X-ray ptychographic measurement system, we investigated the spatial resolution of reconstructed images, robustness to the choice of the hyperparameters and convergence speed of iterative algorithms by comparing the proposed method with ePIE (Maiden & Rodenburg, 2009 ▸) and rPIE (Maiden *et al.*, 2017 ▸). These experiments demonstrated that the proposed method was able to reconstruct higher-spatial-resolution images with half the number of iterations required by ePIE and rPIE while having robustness to hyperparameters. In addition, the proposed method was applied to ptychographic datasets of a Simens star chart and ink toner particles measured at SPring-8 BL24XU, and we confirmed that it was able to successfully reconstruct images from measurement scans with a lower overlap ratio of the illumination regions than is required by ePIE and rPIE.

## 2. Problem formulation

### 2.1. Basic formulation for ptychographic phase retrieval

In ptychography, diffraction intensity patterns are measured by two-dimensionally scanning a specimen (object) at scan intervals where the illuminated regions overlap. The diffraction intensity pattern can be modeled by the squared amplitude of the two-dimensional Fourier transform of the exit wavefield, which corresponds to the multiplication of the probe function and object function. Let 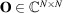 and 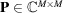 be the two-dimensional object and probe functions, respectively, with *N* > *M*. The *r*th observed diffraction intensity pattern 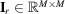 can be represented as 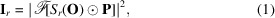
where 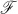 is the two-dimensional Fourier transform, 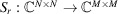 is the sampling operator that extracts the *r*th measurement region, ⊙ denotes element-wise multiplication and | · |^2^ denotes the element-wise squared absolute value.

Ptychographic phase retrieval aims to reconstruct the object **O** and the probe **P** from a set of observed diffraction intensity patterns {**I**_1_,…, **I**_*R*_}. We introduce an auxiliary variable 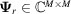 that represents the exit wavefield at the *r*th position and consider the following cost function: 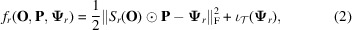
where ∥ · ∥_F_ represents the Frobenius norm and the indicator function 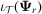, which encodes the constraint, is given by 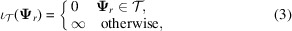

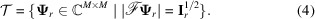
This cost function measures the difference between an estimate of 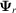, whose modulus of the Fourier transform is constrained to be equal to the observation 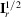, and the exit wavefield calculated from estimates of **O** and **P**. From equation (2), the ptychographic phase-retrieval problem can be formulated as the following optimization problem: 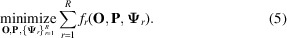
This optimization problem is a basic formulation for ptychographic phase retrieval and leads to some well known algorithms (Chang *et al.*, 2019*a* ▸). For example, ePIE can be interpreted as an algorithm that solves equation (5) by alternating optimization based on the stochastic gradient descent method; DM corresponds to the Douglas–Rachford algorithm for solving equation (5). This formulation makes no special assumptions about scan points and is therefore applicable to various scans *e.g.* a Fermat spiral scan (Huang *et al.*, 2014 ▸), as well as a regular grid scan.

Although some algorithms for solving equation (5) have been proposed (Maiden *et al.*, 2017 ▸, 2012 ▸; Thibault *et al.*, 2009 ▸), finding better solutions is still challenging. This is because the problem in equation (5) involves the product of the optimization variables **O** and **P** and has local minima due to its nonconvexity. To achieve further improvement, we introduce a DNN-based denoiser into the above formulation.

### 2.2. Proposed formulation

We propose a formulation based on a fixed-point constraint (Cohen *et al.*, 2021 ▸). The fixed-point constraint enables us to naturally incorporate a trained DNN into an optimization problem.

A fixed point of an operator *G* is defined as a point **X** satisfying *G*(**X**) = **X**, *i.e.* a point that does not change under the given transformation *G*(**X**) (Combettes & Pesquet, 2020 ▸). Let 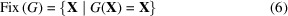
be the set of all fixed points of *G*. If *G* is a trained DNN-based denoiser *D*, then 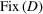 can be interpreted as an approximation of the set of noiseless images. This interpretation is based on the fixed points of an ideal denoiser. The ideal denoiser can remove only noise components from a noisy image and it does not make any changes to noiseless images. Therefore, the set of noiseless images can be modeled by the set of fixed points of the ideal denoiser. Although it is impossible to use such an ideal denoiser in reality, it can be approximated by a denoising DNN that has high denoising performance. Such a denoising DNN is usually trained using an image dataset and additive Gaussian noise because the fixed-point constraint is associated with the maximum *a posteriori* estimation whose likelihood function is Gaussian (Romano *et al.*, 2017 ▸; Cohen *et al.*, 2021 ▸).

According to the above discussion, we propose to formulate ptychographic phase retrieval as a problem of finding a minimizer of equation (5) within the fixed-point set 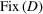, the set of noiseless images approximated by a denoising DNN *D*. This problem can be written as follows: 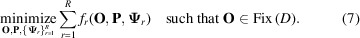
Since **O** obtained by solving equation (7) belongs to 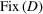, it is expected to be an approximately noiseless reconstruction image.

## 3. Optimization methodology

This section presents the proposed algorithm that approximately solves equation (7) in an alternating-optimization manner. Since it is based on stochastic gradient descent (SGD) (Kleinberg *et al.*, 2018 ▸) and hybrid steepest descent (HSD) algorithms (Yamada & Ogura, 2005 ▸), we first describe them in the following subsections and then introduce the proposed algorithm in Section 3.3.

### 3.1. Stochastic gradient descent algorithm

Consider the problem of minimizing a cost function that has the summation form
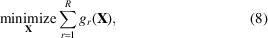
where **X** is a target variable and *g*_*r*_ is a differentiable function associated with the *r*th observation in the dataset. A standard gradient descent (GD) solves (8) by iterating the update 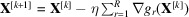, where *k* is the iteration index, η > 0 is the step size and ∇ is the gradient operator. The standard GD algorithm updates using the sum of ∇*g*_*r*_(**X**) with all observations. On the other hand, SGD performs one-by-one updates using ∇*g*_*r*_(**X**) at a single observation. For a given initial value **X**^[0]^, the algorithm of SGD can be written as follows: 
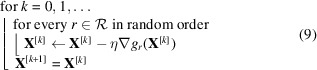
where 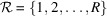. A remarkable feature of SGD is that it can find a better solution than the standard GD for nonconvex optimization problems. This feature has been demonstrated in practical and theoretical studies (Kleinberg *et al.*, 2018 ▸; Keskar *et al.*, 2016 ▸).

### 3.2. Hybrid steepest descent algorithm

The HSD algorithm can handle the following optimization problem with a fixed-point constraint: 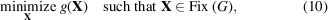
where *G* is an operator and *g* is a differentiable convex function with ξ-Lipschitz gradient ∇*g*. An operator *A* is ξ-Lipschitz if 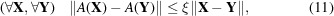
where ∥ · ∥ is the Euclidean norm. If ξ = 1 in equation (11), then *A* is called a nonexpansive operator.

The HSD algorithm iteratively computes the following procedure: 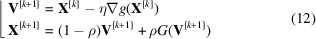
where η > 0 is the step size and ρ > 0 is a hyperparameter. Assuming that *G* is nonexpansive and that 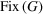
 ∩ 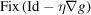 is nonempty (where Id denotes the identity operator), this algorithm converges to a globally optimal solution to equation (10) under the conditions that η ∈ (0, 2/ξ) and ρ ∈ (0, 1/2) (Cohen *et al.*, 2021 ▸). Note that our problem in equation (7) is nonconvex, and thus the global convergence cannot be guaranteed. Even so, it is empirically known that the HSD algorithm can work well for nonconvex problems and perform stable updates in practice with hyperparameters that satisfy the convergence conditions.

### 3.3. Proposed algorithm: PINE

We propose a ptychographic phase-retrieval algorithm, named PINE (ptychographic iterative algorithm with neural denoising engine). It consists of the SGD step in (9) and a denoising step corresponding to the second line of (12) as follows.

The SGD-based step in PINE updates each variable using a single observation and iterates it for all observations in randomly shuffled order. For the *r*th measurement, the update formulas of each variable are derived from the alternating optimization for the problem of minimizing *f*_*r*_.

First, 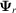 is computed by solving the following subproblem, where **O** and **P** are treated as constants: 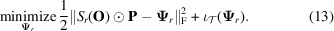
For simplicity, let 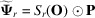. A solution to equation (13) is given by the projection of 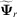 onto 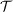 in equation (4) as follows: 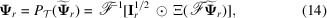
where 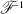 is the two-dimensional inverse Fourier transform and Ξ is the element-wise modified sign function defined by 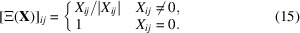
The computation of 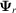 in equation (14) corresponds to the operation that replaces the modulus of the Fourier transform of the exit wave computed from **O** and **P** with the square root of the observed diffraction pattern.

Next, **O** and **P** are updated by the gradient decent for the following subproblems using 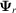 computed by equation (14): 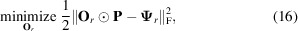

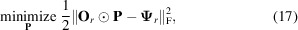
where **O**_*r*_ = *S*_*r*_(**O**). The subproblems are derived by fixing either **O** or **P** and treating the other as the optimization variable in the problem of minimizing *f*_*r*_. By setting the step size parameter η to a value that satisfies the convergence condition of the HSD algorithm, *i.e.* η ∈ (0, 2/ξ), the stochastic gradient descent updates for equations (16) and (17) are obtained as 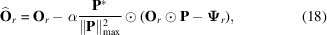

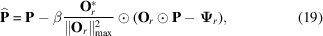
where (·)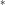 represents the complex conjugate, ∥ · ∥_max_ is the maximum absolute value among all elements of the input variable and α, β ∈ (0, 2) are hyperparameters. The step size parameters of equations (18) and (19) are 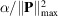 and 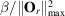, respectively, where 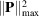 and 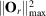 are the Lipschitz constants of the gradients of *f*_*r*_ with respect to **O**_*r*_ and **P**, respectively. The updated object 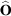 can be obtained by replacing the region of **O** extracted through *S*_*r*_ with 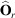, and this operation is denoted by 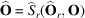.

After the above SGD-based updates for all observations, a denoising step is performed as follows: 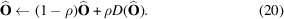
This step corresponds to the second line of the HSD algorithm in (12) and refines the object image using a DNN-based denoiser *D*. The hyperparameter ρ is selected from the open interval (0, 1/2), which satisfies the convergence condition of HSD. We empirically confirmed that tuning of ρ is not important for the final results, and hence we set ρ = 0.49 for all experiments in Section 5.

The entire procedure of PINE is summarized in the following:
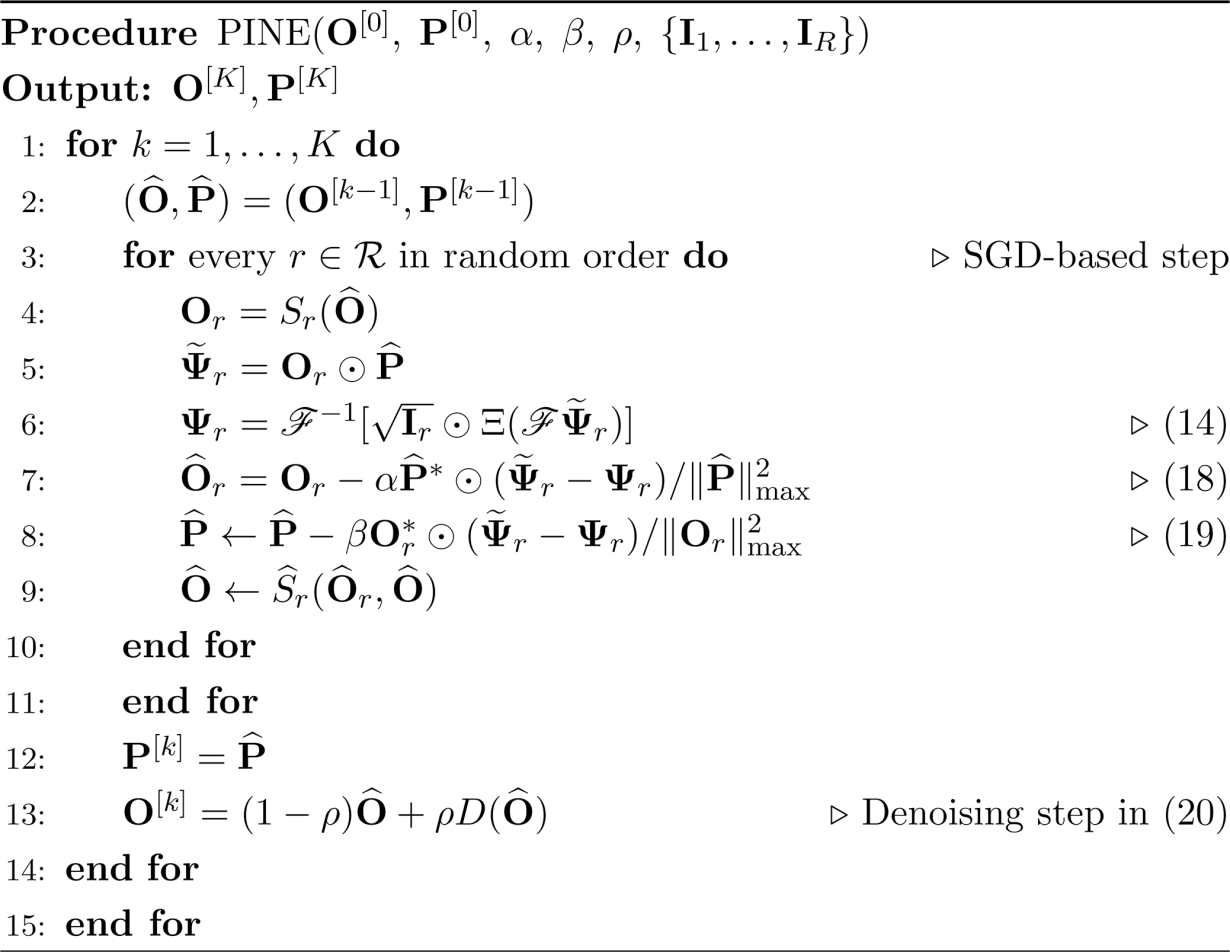
It can be considered as a modified HSD algorithm because the gradient decent update in the first line of (12) is replaced by the SGD-based updates. The key component of PINE is the denoising step using DNN, which provides fast convergence and high-resolution reconstruction images. To make the trial easier, we provide the MATLAB code of the proposed methods at https://github.com/mada-ko/PINE.

PINE includes ePIE as a special case: ρ = 0. Compared with ePIE, PINE requires additional computation due to the denoising step, but it can obtain reconstruction images faster in practice thanks to its faster convergence speed. Furthermore, PINE inherits some useful properties of ePIE: ease of hyperparameter tuning and stable convergence. The hyperparameters of PINE to be tuned are the step sizes α and β for the object and probe updates, which can be adjusted intuitively like for ePIE. The experiments in Section 5 demonstrate that PINE is robust to hyperparameters and experimental conditions and can reconstruct images stably.

## 4. Training of the DNN-based denoiser

In this section, we explain how to construct the DNN-based denoiser *D* used in PINE. Training of the DNN generally requires a large amount of data; however, it is difficult to collect many real object images due to the measurement cost of ptychography. To overcome this difficulty, the proposed method trains *D* using the generated synthetic dataset described in Section 4.1. Moreover, it is necessary to train *D* to be a nonexpansive operator to satisfy the convergence condition of the HSD algorithm. The method of training *D* to be nonexpansive is described in Section 4.2.

### 4.1. Generation of synthetic training dataset

We consider the method of training a DNN-based denoiser without real datasets of object images. One possible choice is to use publicly available image datasets, *e.g.* ImageNet (Deng *et al.*, 2009 ▸), MsCoco (Lin *et al.*, 2014 ▸) *etc*., instead of a real object image dataset. However, the usage of such datasets is often restricted and they can be unavailable because of privacy and ethical concerns (Birhane & Prabhu, 2021 ▸).

For this reason, we adopt an approach that generates synthetic data for training the DNN, called formula-driven supervised learning (FDSL) (Kataoka *et al.*, 2022*a* ▸,*b* ▸; Baradad *et al.*, 2021 ▸). In experimental study of FDSL, Baradad *et al.* (2021 ▸) investigated image-generation models that produce synthetic images from random processes. This study demonstrated that a DNN trained with a synthetic dataset may achieve comparable performance to one trained with a real dataset. Specifically, learning with the dead leaves model provides high performance for specialized tasks, such as the medical or aerial photography domain. According to this result, we generate synthetic training datasets using the dead leaves image models (Baradad *et al.*, 2021 ▸).

**Figure 1:**
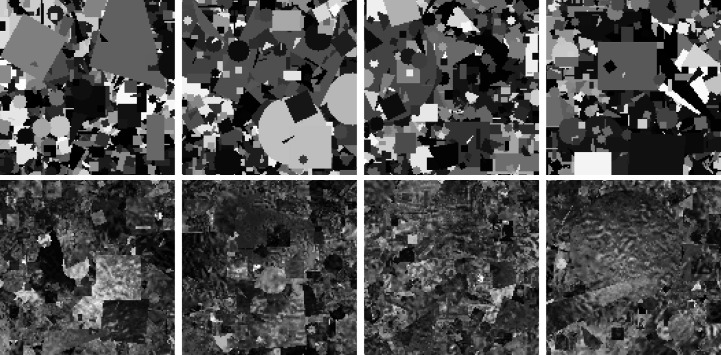
Generated synthetic dataset for training the DNN. The top and bottom rows show examples of images generated by the DL-Diverse model and DL-Textured model, respectively.

The dead leaves model generates synthetic images by randomly positioning simple shapes (circles, triangles and rectangles) until the image canvas is covered. Variants of the dead leaves model have been proposed by Baradad *et al.* (2021 ▸), and we construct the training dataset using two types of models among them: the dead leaves model with rotated multi-size shapes (DL-Diverse) and that with textured shapes (DL-Textured). Fig. 1 ▸ shows examples of images generated by DL-Diverse and DL-Textured. We add white Gaussian noise to the generated images and construct a training dataset for denoising, consisting of pairs of a noisy image and its corresponding ground truth.

### 4.2. Training of the nonexpansive DNN-based denoiser

**Figure 2:**
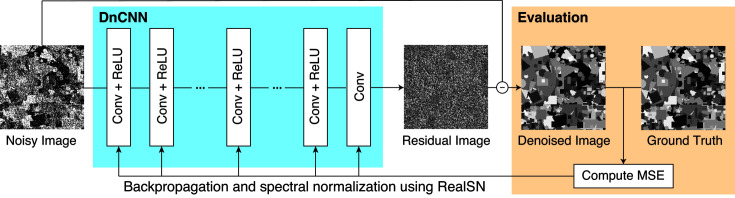
Schematic illustration of training of a nonexpansive DNN-based denoiser.

In this section we explain how to train a nonexpansive DNN-based denoiser. A schematic illustration of the training is shown in Fig. 2 ▸.

We first describe the DNN architecture used in the proposed method. To construct a DNN-based denoiser, we use DnCNN (Zhang *et al.*, 2017 ▸), which is one of the *de facto* standard DNNs for denoising tasks. DnCNN has a simple architecture consisting of convolution (Conv) and rectified linear unit (ReLU) layers, as in Fig. 2 ▸. For the training of DnCNN, the following mean squared error (MSE) is minimized through backpropagation: 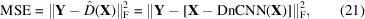
where **X** is a noisy image input to DnCNN, **Y** is the ground truth image, DnCNN(**X**) represents the output of DnCNN and 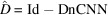. This MSE indicates that DnCNN is trained such that its output is close to the residual **Y** − **X**, *i.e.* added noise. Thus, DnCNN estimates the noise included in input **X** and performs denoising by subtracting it from **X**.

In general, a trained DNN is not a nonexpansive operator, unless a nonexpansiveness constraint is imposed. We exploit RealSN (Ryu *et al.*, 2019 ▸), a spectral normalization method, to constrain DnCNN to be nonexpansive. RealSN computes the Lipschitz constant of the operation in a layer and divides its parameters by the Lipschitz constant, which makes the operation in the layer nonexpansive. Since DnCNN has a simple structure, the entire operation is nonexpansive if the operation in each layer is nonexpansive (Ryu *et al.*, 2019 ▸). We can train nonexpansive DnCNN by applying RealSN to each layer after every weight parameter update through backpropagation.

The DNN-based denoiser constructed in this way is for real-valued images but can perform denoising effectively even for complex-valued objects. Denoising of a complex-valued object **O** with the denoiser 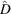 can be represented by 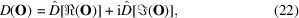
where 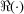 and 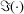 are real and imaginary parts of the input complex number, respectively, and i is the imaginary unit. Another possible way to denoise **O** using 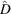 is to independently apply 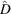 to the amplitude and phase of **O**. However, this approach may suffer from the phase-wrapping problem and is therefore not adopted in this paper.

## 5. Experiments

To evaluate the imaging capability of the proposed method, we conducted experiments with simulated and real data. The simulation mimics the hard X-ray ptychographic measurement system at the imaging station of the Hyogo ID beamline BL24XU at SPring-8 (Takayama *et al.*, 2021 ▸). Since particle dispersions and micro-structured samples are common subjects of observation using ptychography, we chose TiO_2_-particle-filled polymethyl methacrylate film as the specimen in the simulation. The details of the simulation configuration are described in our previous study (Yatabe & Takayama, 2022 ▸). In the experiment, we used two types of simulation data: low-dose data and high-dose data, with diffraction intensities at the origin (*I*_0_) of 10^8^ and 10^10^ photons per pixel, respectively. The diffraction intensity determines the noise level, with lower intensities corresponding to more noise contamination. For the experiment with real data, we used the actual measurements of a Siemens star chart and ink toner particles. The measurement conditions of the data are also described in the previous study (Yatabe & Takayama, 2022 ▸).

To assess the image quality of reconstructed object images for simulation data, we used the Fourier ring correlation (FRC) (Rosenthal & Henderson, 2003 ▸): 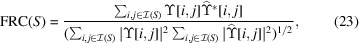
where ϒ is the Fourier transform of a reconstructed object function, 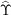 is the Fourier transform of the true object function obtained by simulation and 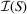 is the set of indices at the ring with radius *r* that corresponds to a given spatial frequency *S*. The closer FRC is to 1, the higher the spatial resolution of the reconstructed image will be. For accurate FRC computation, we performed some preprocessing. We first corrected the amplitude scale and phase offset between true and reconstructed images, and then they were subpixel-aligned using the phase-only correlation method. To eliminate the influence of the peripheral area of the image where the illumination intensity is insufficient, *i.e.* the total number of irradiated photons is less than 1% of the maximum, the true and reconstructed images were set to a vacuum. In these simulation data, the amplitude transmittance was close to 1, and the peripheral area was small compared with the comparison region, so edge effects can be ignored.

For the training of the DNN, we constructed a synthetic dataset that consists of 500 images for each of two image models, DL-Diverse and DL-Textured, *i.e.* 1000 images in total. The generated images were 8 bit images of size 128 × 128, and their intensity range was [0, 255]. Gaussian noise with a standard deviation σ = 15 was added to them. When training the denoiser, we normalized these images to the range [0, 1]. The dataset generation and the training of the DNN were implemented in Python 3.10 and were run on a 2.0 GHz Intel Core i9-13900 processor with 32 GB RAM and NVIDIA GeForce RTX 3060. The computation time for generating these 1000 images was 6 min. We trained the DNN-based denoiser for 50 epochs, which required less than 8 h.

### 5.1. Performance comparison with simulation data

To perform a reasonable assessment, we adopt ePIE and rPIE as comparison methods, because comparison with algorithms derived in significantly different ways from the proposed method may make the effect of the DNN-based denoiser unclear. For all the algorithms, the initial probe estimate was set to a bump function with support approximately the same size as the simulation probe, and the initial object estimate was set to a vacuum (*i.e.* 1). The sequences to access the indices of diffraction patterns in random order were kept consistent among the different algorithms.

All algorithms have hyperparameters α and β, which were set to the best values among 100 different combinations. For ePIE and PINE, both α and β were selected from {0.1*m* | *m* = 1, 2,…, 10}; for rPIE, α and β were selected from {0.05*m* | *m* = 1, 2,…, 10} and {0.5*m* | *m* = 1, 2,…, 10}, respectively. The range for rPIE was determined according to the suggested hyperparameter ranges described by Maiden *et al.* (2017 ▸). The selected hyperparameter combinations (α, β) in the low-dose data were (0.3, 0.2) for ePIE, (0.15, 5) for rPIE and (0.5, 0.1) for PINE; those in the high-dose data were (0.4, 0.8) for ePIE, (0.1, 3.0) for rPIE and (0.8, 0.5) for PINE. Robustness to hyperparameter selection is discussed in Section 5.2.

**Figure 3:**
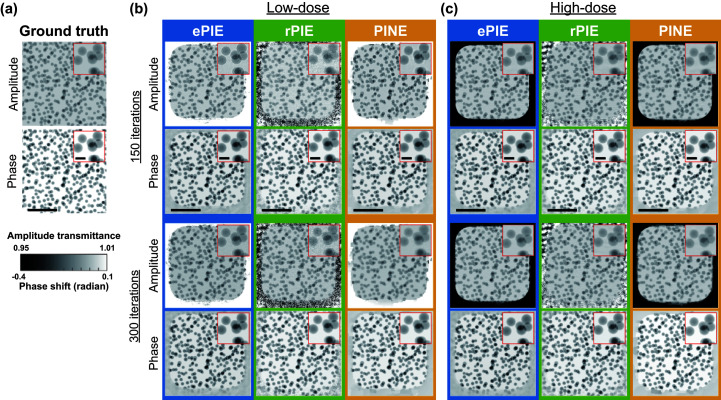
Comparison of the images reconstructed from the simulated data, demonstrating the convergence properties. (*a*) The ground truth of the amplitude and phase images. (*b*) The reconstructed images for low-dose simulations, and (*c*) those for high-dose simulations. The first and second rows show the amplitude and phase of the constructed images after 150 iterations, and the third and fourth rows show those after 300 iterations, for ePIE, rPIE and PINE. The insets show a magnified area. The black bars in the second row indicate 4 µm, and those in the insets are 0.5 µm.

The reconstructed images are shown in Fig. 3 ▸. As can be seen in Fig. 3 ▸(*b*), for low-dose data PINE obtained higher-quality images, *i.e.* closer to the ground truth images, compared with the other methods even when the number of iterations was 150. On the other hand, in the reconstructed amplitude image of ePIE, we can find artifacts that enhance the contour of particles in white, by comparing the reconstructed and ground truth images.

This artifact is known as the refractive contrast (Born & Wolf, 1999 ▸; Paganin *et al.*, 2002 ▸; Snigirev *et al.*, 1995 ▸) and often occurs when the algorithm does not converge sufficiently. The reconstructed image of rPIE for low-dose data was contaminated with more noise than those of the other methods. From Fig. 3 ▸(*c*), it is found that for high-dose data PINE and rPIE were able to successfully reconstruct the images even with 150 iterations, while ePIE obtained an image containing refractive contrast artifacts with 150 iterations.

**Figure 4:**
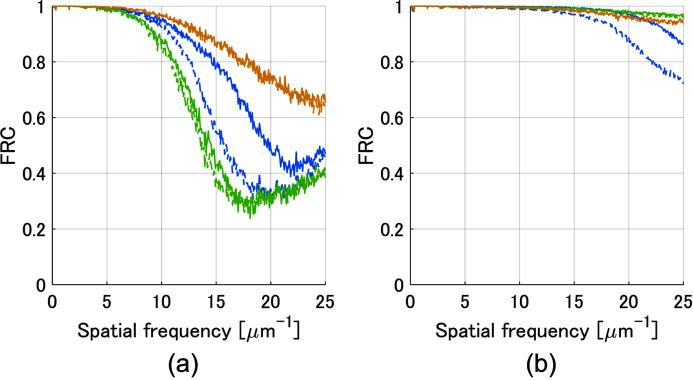
Convergence properties of each algorithm evaluated with FRC curves. (*a*) FRC curves for low-dose simulation, and (*b*) those for high-dose simulation. The blue, green and yellow lines correspond to ePIE, rPIE and PINE, respectively. The dashed and solid lines represent the results of the 150 and 300 iterations, respectively.

The FRC of the reconstructed images for each algorithm is shown in Fig. 4 ▸, where the blue, green and yellow lines represent ePIE, rPIE and PINE, respectively, and the solid and dashed lines correspond to the results with 150 and 300 iterations. For low-dose data, PINE with 150 iterations outperformed the other methods with 300 iterations, and for high-dose data, PINE was able to achieve higher spatial resolution than ePIE and comparable resolution to rPIE. This indicates that PINE can obtain higher-resolution images with half the number of iterations required by ePIE and rPIE, especially for low-dose data.

### 5.2. Robustness to hyperparameter selection

The practical application of ptychography requires a phase-retrieval algorithm that is robust to the choice of hyperparameters and can be intuitively tuned. One of the algorithms that meet these requirements is ePIE, which is used in many practical applications such as multi-slice 3D imaging (Maiden *et al.*, 2012 ▸), measurement position correction (Zhang *et al.*, 2013 ▸; Tripathi *et al.*, 2014 ▸) and multi-mode reconstruction (Li *et al.*, 2016 ▸). To evaluate the robustness of the algorithms to the choice of hyperparameters, we examined how the changes in hyperparameters affect the FRC of the reconstructed image and compared PINE with ePIE and rPIE.

**Figure 5:**
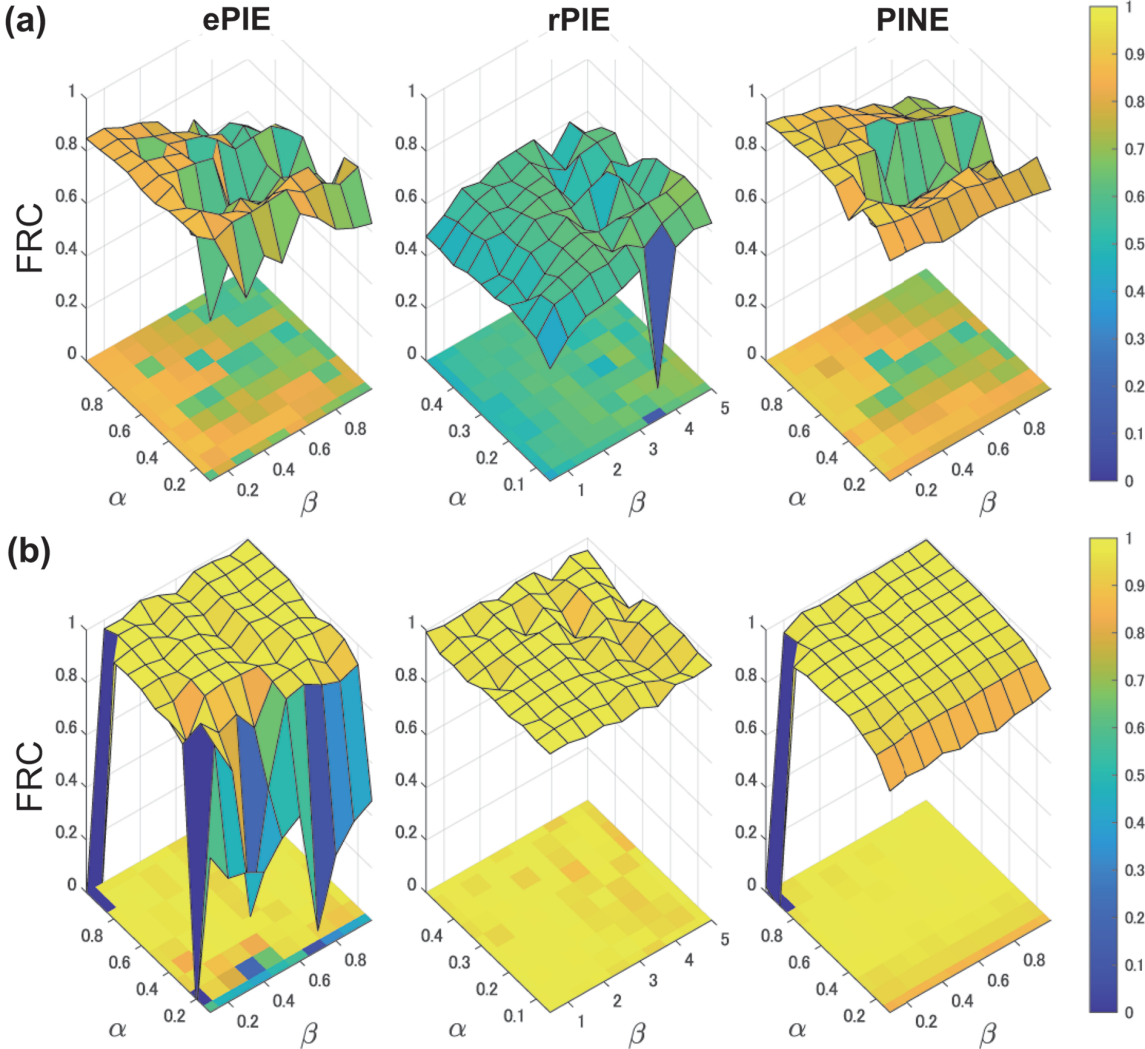
Robustness of each algorithm against the hyperparameters. FRC at a spatial frequency of 12.5 µm^−1^, half of the maximum spatial frequency, is mapped for 100 different combinations of the hyperparameters α and β. (*a*) FRC for low-dose simulation, and (*b*) FRC for high-dose simulation. They were computed from the reconstructed images after 300 iterations.

Fig. 5 ▸ shows the FRC at the spatial frequency of 12.5 µm^−1^, which is half of the maximum spatial frequency, for the 100 hyperparameter combinations described in Section 5.1. For all algorithms, the FRC was computed from the reconstructed image after 300 iterations. PINE was able to stably achieve higher spatial resolution for both low-dose and high-dose data and for most hyperparameter combinations. This demonstrates that the proposed method can determine appropriate hyperparameters in a shorter time than ePIE. The behavior of FRC for hyperparameters in PINE is similar to that in ePIE. This can be interpreted as PINE inheriting the good properties of ePIE with respect to hyperparameters because it includes ePIE as a special case. This result implies that PINE can be used in place of ePIE and could improve imaging capability in many practical applications.

### 5.3. Comparison of computational time

The convergence speed of the algorithm is important for ptychography measurements to be conducted without delay. The convergence speed and computational time of PINE were compared with those of ePIE and rPIE. All methods were implemented in MATLAB R2023a and run on a 2.0 GHz Intel Core i9-13900 processor with 32 GB RAM. We recorded the computational time of each algorithm for simulated data. The hyperparameters for each method were the same as in Section 5.1.

**Figure 6:**
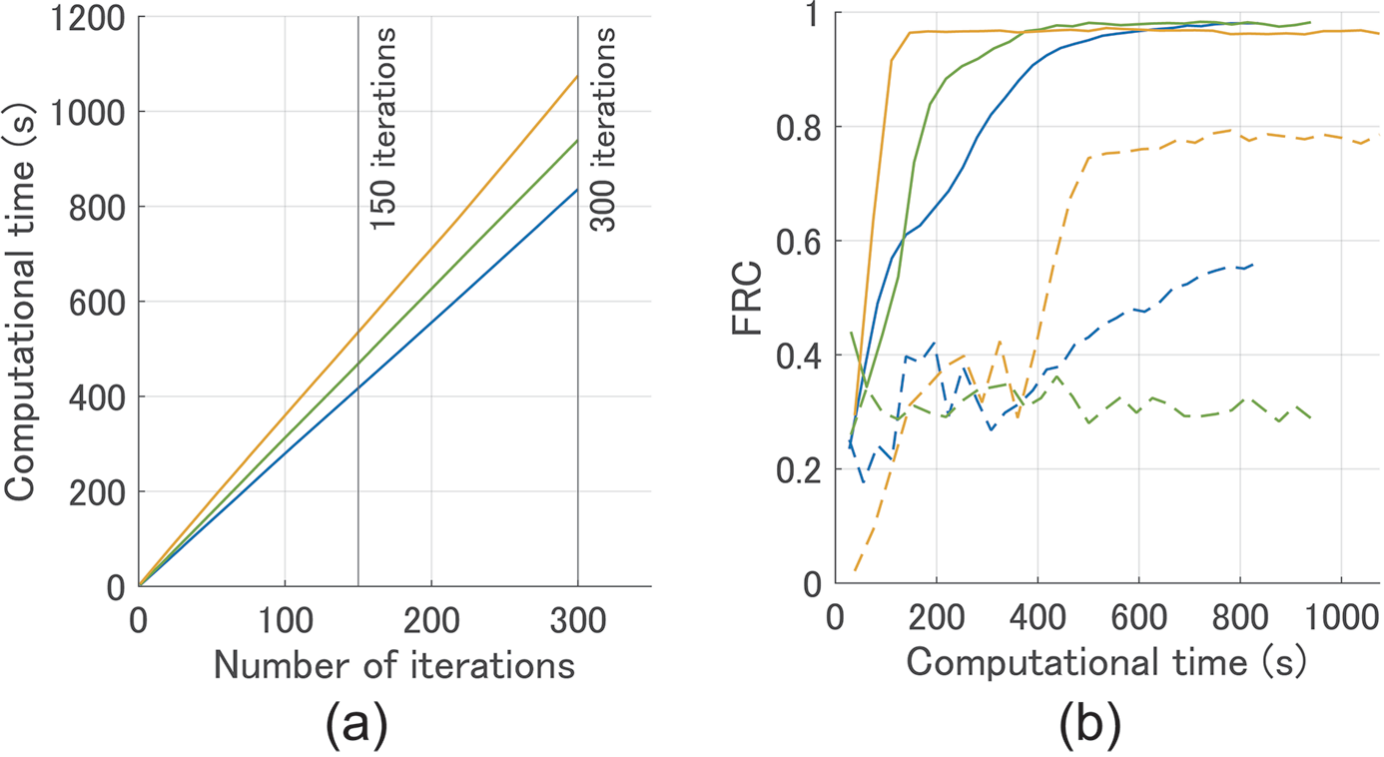
Comparison of computational time. (*a*) Computational time (s) versus the number of iterations. (*b*) FRC at a spatial frequency of 18.75 µm^−1^ versus computational time (s). The blue, green and yellow lines correspond to ePIE, rPIE and PINE, respectively. The dashed and solid lines in (*b*) represent the results for the low-dose and high-dose data, respectively.

Fig. 6 ▸ shows a comparison of the computational time. The blue, green and yellow lines correspond to ePIE, rPIE and PINE, respectively. The computational time (s) against the number of iterations is shown in Fig. 6 ▸(*a*). The average computational time per iteration was 2.8 s for ePIE, 3.1 s for rPIE and 3.6 s for PINE. Since PINE requires additional processing (*i.e.* the computation of the denoising step) compared with the other methods, it took slightly more computational time for each iteration. However, PINE can obtain higher-resolution reconstruction images with fewer iterations than the other methods in practice. This can be confirmed from Fig. 6 ▸(*b*). This figure shows the FRC at a spatial frequency of 18.75 µm^−1^ against computational time (s), where 18.75 µm^−1^ corresponds to three-quarters of the maximum spatial frequency, and the dashed and solid lines represent the results for the low-dose and high-dose data, respectively. For high-dose simulation, the time it took to achieve the FRC of over 0.95 was 500 s for ePIE, 375 s for rPIE and 147 s for PINE. These results demonstrate that inserting a denoiser does not impose high computational costs and can improve convergence speed.

### 5.4. Results for real data

In general, imaging capability improves as the number of measurements of diffraction patterns increases (Bunk *et al.*, 2008 ▸). However, measurement scans with a high overlap ratio cause serious radiation damage to a specimen in some cases of actual ptychographic measurement. Therefore, it is desirable for the phase-retrieval algorithms to reconstruct a high-resolution image using fewer measurements. To evaluate imaging capability for different overlap ratios, we decimated the observed diffraction patterns of the real data, the Siemens star chart and the ink toner particles. We set the hyperparameters (α, β) = (1, 0.1) for both ePIE and PINE, and (α, β) = (0.1, 1) for rPIE.

**Figure 7:**
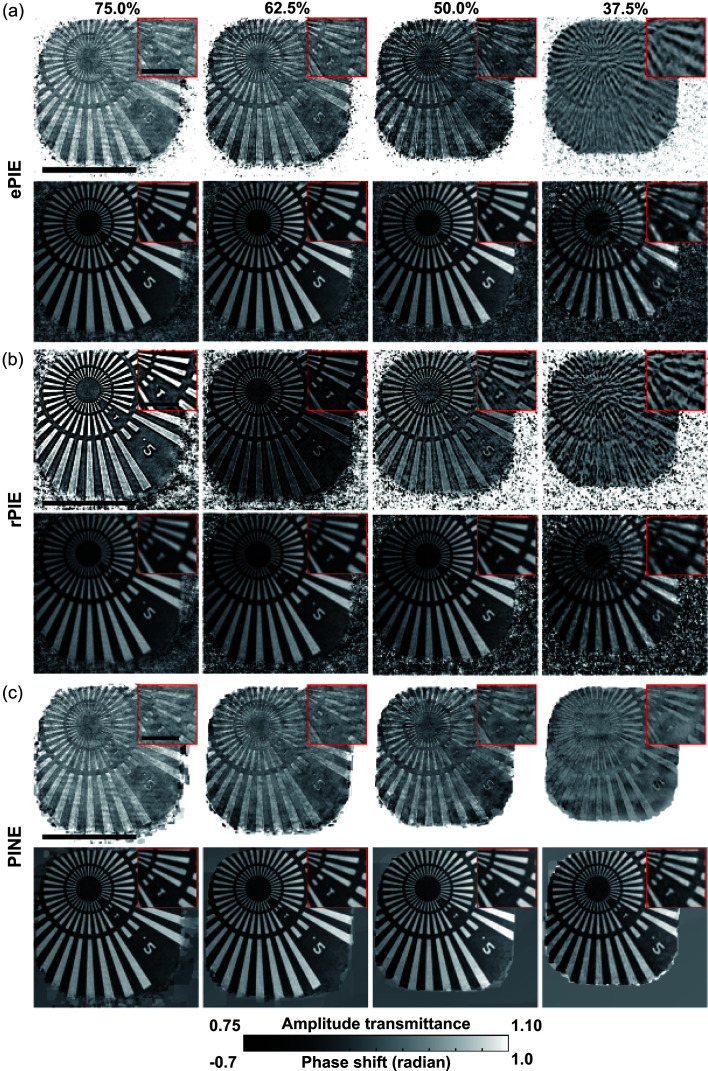
Reconstructed images of the Siemens star chart data with different overlap ratios. (*a*)–(*c*) Amplitude (upper) and phase (lower) images reconstructed by (*a*) ePIE, (*b*) rPIE and (*c*) PINE, with overlap ratios indicated at the top. The black bars in the main images represent 4 µm, and those in the insets are 1 µm.

**Figure 8:**
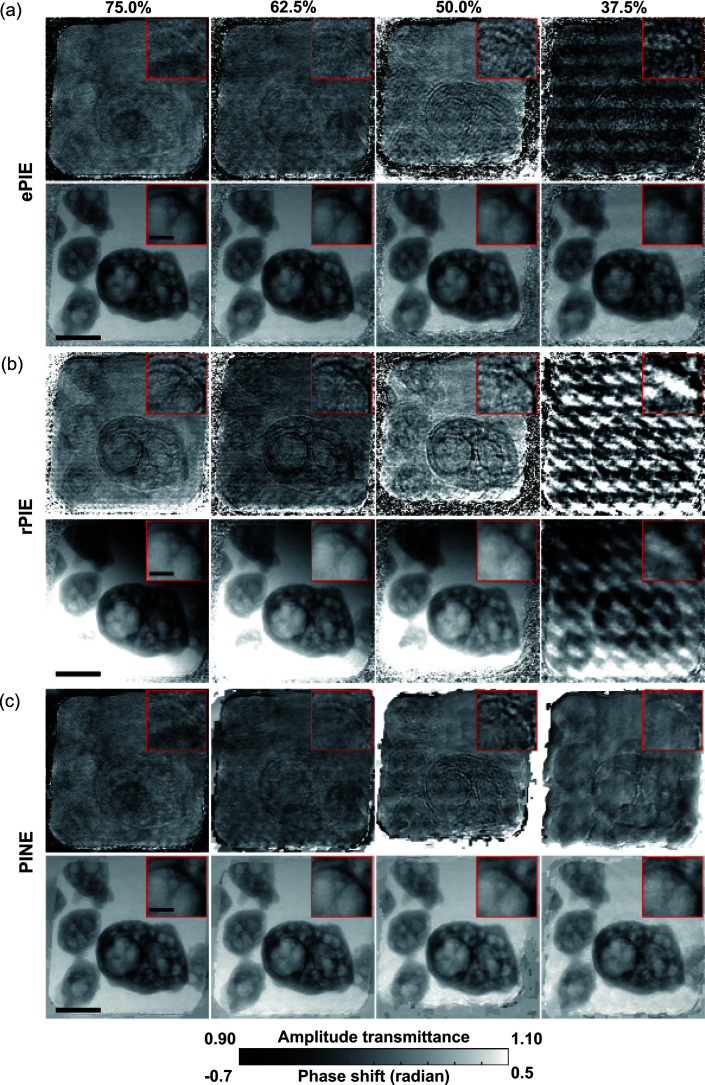
Reconstructed images of the ink toner particles with different overlap ratios. (*a*)–(*c*) Amplitude (upper) and phase (lower) images reconstructed by (*a*) ePIE, (*b*) rPIE and (*c*) PINE, with overlap ratios indicated at the top. The black bars in the main images represent 4 µm, and those in the insets are 1 µm.

Fig. 7 ▸ shows the reconstructed images of the Siemens star chart data with different overlap ratios. As can be seen in this figure, ePIE and rPIE suffered from degradation of the amplitude images with a 50.0% overlap ratio and severe degradation of both amplitude and phase images with a 37.5% overlap ratio. In the amplitude images reconstructed by rPIE, the contour of the radial slit pattern is enhanced brighter on the slit hole side (white area) and darker on the tantalum foil side (dark area), which is characteristic of refraction contrast (Snigirev *et al.*, 1995 ▸) as mentioned in Section 5.1 and suggests that this is an artifact due to the slight shift of the reconstruction plane downstream in the optical axis direction. On the other hand, PINE obtained similar amplitude and phase images for all overlap ratios and successfully reconstructed images even for a 37.5% overlap ratio. Fig. 8 ▸ shows the reconstructed images of the ink toner particles with different overlap ratios. Similarly to Fig. 7 ▸, even for the overlap ratio of 37.5%, PINE was able to reconstruct images closer to the reconstructed one at the overlap ratio of 75.0%, while severe degradation was observed in the amplitude images for ePIE and both the amplitude and phase images for rPIE. These experimental results demonstrate that PINE is more robust to the change in overlap ratio than the other methods.

## 6. Conclusion

This paper has proposed the ptychographic phase-retrieval algorithm called PINE, which combines model-based and DNN-based approaches. The proposed method incorporates a denoising DNN into an iterative algorithm derived from the measurement model and improves both the imaging capability and the robustness to changes in experimental conditions. The DNN is trained with synthetic datasets generated by the FDSL techniques to avoid the difficulty of collecting many measured specimen images for training. Experimental results using both simulated and real data showed that PINE successfully reconstructed high-spatial-resolution images with half the number of iterations required by ePIE and rPIE while inheriting the favorable properties of ePIE, such as stable convergence and robustness to hyperparameters. The idea of using a denoising DNN to assist an iterative algorithm in finding better reconstruction images could be easily incorporated into other methods like DM and RAAR, which could be a direction of future work. Our future work includes validation of such extended methods, improvements of the denoising performance of nonexpansive DNNs, GPU-based implementation of the proposed method, and applying PINE to practical applications such as 3D imaging through a multi-slice approach and measurement position correction.

## References

[bb1] Baradad, M., Wulff, J., Wang, T., Isola, P. & Torralba, A. (2021). *Advances in Neural Information Processing Systems 34 (NeurIPS 2021)*, edited by M. Ranzato, A. Beygelzimer, Y. Dauphin, P. S. Liang & J. Wortman Vaughan, pp. 2556–2569. Red Hook: Curran Associates.

[bb2] Barbastathis, G., Ozcan, A. & Situ, G. (2019). *Optica*, **6**, 921–943.

[bb3] Birhane, A. & Prabhu, V. U. (2021). *2021 IEEE Winter Conference on Applications of Computer Vision (WACV)*, pp. 1536–1546. Piscataway: IEEE.

[bb4] Born, M. & Wolf, E. (1999). *Principles of Optics: Electromagnetic Theory of Propagation, Interference and Diffraction of Light*, 7th ed. Cambridge University Press.

[bb5] Bostan, E., Heckel, R., Chen, M., Kellman, M. & Waller, L. (2020). *Optica*, **7**, 559–562.

[bb6] Bunk, O., Dierolf, M., Kynde, S., Johnson, I., Marti, O. & Pfeiffer, F. (2008). *Ultramicroscopy*, **108**, 481–487. 10.1016/j.ultramic.2007.08.003 17764845

[bb7] Chang, H., Enfedaque, P. & Marchesini, S. (2019*a*). *SIAM J. Imaging Sci.* **12**, 153–185.

[bb8] Chang, H., Enfedaque, P., Zhang, J., Reinhardt, J., Enders, B., Yu, Y.-S., Shapiro, D., Schroer, C. G., Zeng, T. & Marchesini, S. (2019*b*). *Opt. Express*, **27**, 10395–10418. 10.1364/OE.27.010395 31052900

[bb9] Cherukara, M. J., Zhou, T., Nashed, Y., Enfedaque, P., Hexemer, A., Harder, R. J. & Holt, M. V. (2020). *Appl. Phys. Lett.* **117**, 044103.

[bb10] Cohen, R., Elad, M. & Milanfar, P. (2021). *SIAM J. Imaging Sci.* **14**, 1374–1406.

[bb11] Combettes, P. L. & Pesquet, J.-C. (2020). *SIAM J. Math. Data Sci.* **2**, 529–557.

[bb12] De Caro, L., Altamura, D., Arciniegas, M., Siliqi, D., Kim, M. R., Sibillano, T., Manna, L. & Giannini, C. (2016). *Sci. Rep.* **6**, 19397. 10.1038/srep19397 PMC4726119 26775682

[bb13] Deng, J., Dong, W., Socher, R., Li, L.-J., Li, K. & Fei-Fei, L. (2009). *2009 IEEE Conference on Computer Vision and Pattern Recognition*, pp. 248–255. Piscataway: IEEE.

[bb14] Deng, J., Lo, Y. H., Gallagher-Jones, M., Chen, S., Pryor, A., Jin, Q., Hong, Y. P., Nashed, Y. S. G., Vogt, S., Miao, J. & Jacobsen, C. (2018). *Sci. Adv.* **4**, eaau4548. 10.1126/sciadv.aau4548 PMC6214637 30406204

[bb15] Deng, J., Preissner, C., Klug, J. A., Mashrafi, S., Roehrig, C., Jiang, Y., Yao, Y., Wojcik, M., Wyman, M. D., Vine, D., Yue, K., Chen, S., Mooney, T., Wang, M., Feng, Z., Jin, D., Cai, Z., Lai, B. & Vogt, S. (2019). *Rev. Sci. Instrum.* **90**, 083701. 10.1063/1.5103173 31472643

[bb16] Gao, Z., Holler, M., Odstrcil, M., Menzel, A., Guizar-Sicairos, M. & Ihli, J. (2020). *Chem. Commun.* **56**, 13373–13376. 10.1039/d0cc06101h 33030473

[bb17] Grote, L., Seyrich, M., Döhrmann, R., Harouna-Mayer, S. Y., Mancini, F., Kaziukenas, E., Fernandez-Cuesta, I. A., Zito, C., Vasylieva, O., Wittwer, F., Odstrčzil, M., Mogos, N., Landmann, M., Schroer, C. G. & Koziej, D. (2022). *Nat. Commun.* **13**, 4971. 10.1038/s41467-022-32373-2 PMC9424245 36038564

[bb18] Guizar-Sicairos, M. & Fienup, J. R. (2008). *Opt. Express*, **16**, 7264–7278. 10.1364/oe.16.007264 18545432

[bb19] Hesse, R., Luke, D. R., Sabach, S. & Tam, M. K. (2015). *SIAM J. Imaging Sci.* **8**, 426–457.

[bb20] Hoidn, O., Mishra, A. A. & Mehta, A. (2023). *Sci. Rep.* **13**, 22789. 10.1038/s41598-023-48351-7 PMC10733394 38123573

[bb21] Huang, X., Yan, H., Harder, R., Hwu, Y., Robinson, I. K. & Chu, Y. S. (2014). *Opt. Express*, **22**, 12634–12644. 10.1364/OE.22.012634 24921380

[bb22] Ishiguro, N., Kaneko, F., Abe, M., Takayama, Y., Yoshida, J., Hoshino, T., Takazawa, S., Uematsu, H., Sasaki, Y., Okawa, N., Takahashi, K., Takizawa, H., Kishimoto, H. & Takahashi, Y. (2024). *Appl. Phys. Expr.* **17**, 052006.

[bb23] Jo, Y., Cho, H., Lee, S. Y., Choi, G., Kim, G., Min, H. & Park, Y. (2019). *IEEE J. Sel. Top. Quantum Electron.* **25**, 1–14.

[bb24] Kataoka, H., Hayamizu, R., Yamada, R., Nakashima, K., Takashima, S., Zhang, X., Martinez-Noriega, E. J., Inoue, N. & Yokota, R. (2022*a*). *2022 IEEE/CVF Conference on Computer Vision and Pattern Recognition (CVPR)*, pp. 21232–21241. Piscataway: IEEE.

[bb25] Kataoka, H., Okayasu, K., Matsumoto, A., Yamagata, E., Yamada, R., Inoue, N., Nakamura, A. & Satoh, Y. (2022*b*). *Int. J. Comput. Vis.* **130**, 990–1007.

[bb26] Keskar, N. S., Mudigere, D., Nocedal, J., Smelyanskiy, M. & Tang, P. T. P. (2016). *arXiv*:1609.04836.

[bb27] Kleinberg, B., Li, Y. & Yuan, Y. (2018). *Proc. Mach. Learn. Res.* **80**, 2698–2707.

[bb28] Li, P., Edo, T., Batey, D., Rodenburg, J. & Maiden, A. (2016). *Opt. Express*, **24**, 9038–9052. 10.1364/OE.24.009038 27137333

[bb29] Li, S., Deng, M., Lee, J., Sinha, A. & Barbastathis, G. (2018). *Optica*, **5**, 803–813.

[bb30] Lin, T.-Y., Maire, M., Belongie, S., Hays, J., Perona, P., Ramanan, D., Dollár, P. & Zitnick, C. L. (2014). *Computer Vision – ECCV 2014*, edited by D. Fleet, T. Pajdla, B. Schiele & T. Tuytelaars, pp. 740–755. Cham: Springer.

[bb31] Luke, D. R. (2005). *Inverse Probl.* **21**, 37–50.

[bb32] Maiden, A., Johnson, D. & Li, P. (2017). *Optica*, **4**, 736–745.

[bb33] Maiden, A. M., Humphry, M. J. & Rodenburg, J. M. (2012). *J. Opt. Soc. Am. A*, **29**, 1606–1614. 10.1364/JOSAA.29.001606 23201876

[bb34] Maiden, A. M. & Rodenburg, J. M. (2009). *Ultramicroscopy*, **109**, 1256–1262. 10.1016/j.ultramic.2009.05.012 19541420

[bb35] Marchesini, S., Krishnan, H., Daurer, B. J., Shapiro, D. A., Perciano, T., Sethian, J. A. & Maia, F. R. N. C. (2016). *J. Appl. Cryst.* **49**, 1245–1252.

[bb36] Nguyen, T., Xue, Y., Li, Y., Tian, L. & Nehmetallah, G. (2018). *Opt. Express*, **26**, 26470–26484. 10.1364/OE.26.026470 30469733

[bb37] Paganin, D., Mayo, S. C., Gureyev, T. E., Miller, P. R. & Wilkins, S. W. (2002). *J. Microsc.* **206**, 33–40. 10.1046/j.1365-2818.2002.01010.x 12000561

[bb38] Pattammattel, A., Tappero, R., Ge, M., Chu, Y. S., Huang, X., Gao, Y. & Yan, H. (2020). *Sci. Adv.* **6**, eabb3615. 10.1126/sciadv.abb3615 PMC11206466 32917679

[bb39] Polo, C. C., Pereira, L., Mazzafera, P., Flores-Borges, D. N. A., Mayer, J. L. S., Guizar-Sicairos, M., Holler, M., Barsi-Andreeta, M., Westfahl, H. & Meneau, F. (2020). *Sci. Rep.* **10**, 6023. 10.1038/s41598-020-63093-6 PMC7138792 32265529

[bb40] Rivenson, Y., Zhang, Y., Günaydın, H., Teng, D. & Ozcan, A. (2018). *Light Sci. Appl.* **7**, 17141. 10.1038/lsa.2017.141 PMC6060068 30839514

[bb41] Rodenburg, J. M. & Faulkner, H. M. L. (2004). *Appl. Phys. Lett.* **85**, 4795–4797.

[bb42] Romano, Y., Elad, M. & Milanfar, P. (2017). *SIAM J. Imaging Sci.* **10**, 1804–1844.

[bb43] Rosenthal, P. B. & Henderson, R. (2003). *J. Mol. Biol.* **333**, 721–745. 10.1016/j.jmb.2003.07.013 14568533

[bb44] Ryu, E., Liu, J., Wang, S., Chen, X., Wang, Z. & Yin, W. (2019). *Proc. Mach. Learn. Res* **97**, 5546–5557.

[bb45] Seifert, J., Shao, Y., van Dam, R., Bouchet, D., van Leeuwen, T. & Mosk, A. P. (2023). *Opt. Lett.* **48**, 6027–6030. 10.1364/OL.502344 37966780

[bb46] Shahmoradian, S. H., Tsai, E. H. R., Diaz, A., Guizar-Sicairos, M., Raabe, J., Spycher, L., Britschgi, M., Ruf, A., Stahlberg, H. & Holler, M. (2017). *Sci. Rep.* **7**, 6291. 10.1038/s41598-017-05587-4 PMC5524705 28740127

[bb47] Shi, X., Burdet, N., Chen, B., Xiong, G., Streubel, R., Harder, R. & Robinson, I. K. (2019). *Appl. Phys. Rev.* **6**, 011306.

[bb48] Sinha, A., Lee, J., Li, S. & Barbastathis, G. (2017). *Optica*, **4**, 1117–1125.

[bb49] Snigirev, A., Snigireva, I., Kohn, V., Kuznetsov, S. & Schelokov, I. (1995). *Rev. Sci. Instrum.* **66**, 5486–5492.

[bb50] Sun, T., Sun, G., Yu, F., Mao, Y., Tai, R., Zhang, X., Shao, G., Wang, Z., Wang, J. & Zhou, J. (2021). *ACS Nano*, **15**, 1475–1485. 10.1021/acsnano.0c08891 33356135

[bb51] Suzuki, A., Shimomura, K., Hirose, M., Burdet, N. & Takahashi, Y. (2016). *Sci. Rep.* **6**, 35060. 10.1038/srep35060 PMC5062076 27734961

[bb52] Takayama, Y., Fukuda, K., Kawashima, M., Aoi, Y., Shigematsu, D., Akada, T., Ikeda, T. & Kagoshima, Y. (2021). *Commun. Phys.* **4**, 48.

[bb53] Thibault, P., Dierolf, M., Bunk, O., Menzel, A. & Pfeiffer, F. (2009). *Ultramicroscopy*, **109**, 338–343. 10.1016/j.ultramic.2008.12.011 19201540

[bb54] Thibault, P. & Guizar-Sicairos, M. (2012). *New J. Phys.* **14**, 063004.

[bb55] Tripathi, A., McNulty, I. & Shpyrko, O. G. (2014). *Opt. Express*, **22**, 1452–1466. 10.1364/OE.22.001452 24515152

[bb56] Uematsu, H., Ishiguro, N., Abe, M., Takazawa, S., Kang, J., Hosono, E., Nguyen, N. D., Dam, H. C., Okubo, M. & Takahashi, Y. (2021). *J. Phys. Chem. Lett.* **12**, 5781–5788. 10.1021/acs.jpclett.1c01445 34137620

[bb57] Wu, J., Zhu, X., West, M. M., Tyliszczak, T., Shiu, H.-W., Shapiro, D., Berejnov, V., Susac, D., Stumper, J. & Hitchcock, A. P. (2018). *J. Phys. Chem. C*, **122**, 11709–11719.

[bb58] Yamada, I. & Ogura, N. (2005). *Numer. Funct. Anal. Optim.* **25**, 619–655.

[bb59] Yatabe, K. & Takayama, Y. (2022). *J. Appl. Cryst.* **55**, 978–992.

[bb60] Zhang, F., Peterson, I., Vila-Comamala, J., Diaz, A., Berenguer, F., Bean, R., Chen, B., Menzel, A., Robinson, I. K. & Rodenburg, J. M. (2013). *Opt. Express*, **21**, 13592–13606. 10.1364/OE.21.013592 23736612

[bb61] Zhang, K., Zuo, W., Chen, Y., Meng, D. & Zhang, L. (2017). *IEEE Trans. Image Process.* **26**, 3142–3155. 10.1109/TIP.2017.2662206 28166495

[bb62] Zhou, L., Song, J., Kim, J. S., Pei, X., Huang, C., Boyce, M., Mendonça, L., Clare, D., Siebert, A., Allen, C. S., Liberti, E., Stuart, D., Pan, X., Nellist, P. D., Zhang, P., Kirkland, A. I. & Wang, P. (2020). *Nat. Commun.* **11**, 2773. 10.1038/s41467-020-16391-6 PMC7265480 32487987

